# Alzheimer-Type Cerebral Amyloidosis in the Context of HIV Infection: Implications for a Proposed New Treatment Approach

**DOI:** 10.1007/s11481-024-10126-w

**Published:** 2024-06-03

**Authors:** Ronald J. Ellis, Shibangi Pal, Cristian L. Achim, Erin Sundermann, David J. Moore, Virawudh Soontornniyomkij, Howard Feldman

**Affiliations:** 1 Department of Neuroscience, University of California, San Diego, CA USA; 2 Department of Psychiatry, University of California, San Diego, CA USA

**Keywords:** Amyloid, HIV, Alzheimer’s disease, Reverse transcriptase inhibitors, Cerebral amyloidosis

## Abstract

Reverse transcriptase inhibitors (RTIs) are currently broadly prescribed for the treatment of HIV infection but are also thought to prevent Alzheimer’s disease (AD) progression by protecting against amyloidosis. Our study evaluates the hypothesis that reverse transcriptase inhibitors protect against Alzheimer-type brain amyloidogenesis in the context of HIV infection. We compiled a case series of participants from a prospective study of the neurological consequences of HIV infection at the HIV Neurobehavioral Research Program (HNRP) who had serial neuropsychological and neurological assessments and were on RTIs. Two participants had gross and microscopic examination and immunohistochemistry of the brain at autopsy; one was assessed clinically for Alzheimer’s disease by cerebrospinal fluid (CSF) analysis of phosphorylated-Tau, Total-Tau and Aβ42. Additionally, a larger cohort of 250 autopsied individuals was evaluated for presence of amyloid plaques, Tau, and related pathologies. Three older, virally suppressed individuals with HIV who had long-term treatment with RTIs were included in analyses. Two cases demonstrated substantial cerebral amyloid deposition at autopsy. The third case met clinical criteria for AD based on a typical clinical course and CSF biomarker profile. In the larger cohort of autopsied individuals, the prevalence of cerebral amyloidosis among people with HIV (PWH) was greater for those on RTIs. Our study showed that long-term RTI therapy did not protect against Alzheimer-type brain amyloidogenesis in the context of HIV infection in these patients. Given the known toxicities of RTIs, it is premature to recommend them to individuals at risk or with Alzheimer’s disease who do not have HIV infection.

## Introduction

HIV promotes brain amyloid deposition through a variety of mechanisms, including neuroinflammation (Andras and Toborek 2013). Thus, infected microglia produce the inflammatory cytokines tumor necrosis factor-α (TNFα) and interleukin 1β (IL1β), which in turn increase APP transcription and stimulate increased cleavage of APP by β and γ secretases to release amyloid that can be deposited in brain tissue (Haughey et al. 2010). In a recent report, the authors proposed that a widely available class of anti-HIV drugs (antiretrovirals, ARVs) might protect against the development of cerebral amyloidosis and described a novel mechanism through which this protection might be achieved (Lee et al. 2018). This mechanism involves somatic recombination of amyloid precursor (APP) gene transcripts in neurons, leading to the expression of mutated amyloid proteins. Reverse transcriptases (RTs) encoded by human endogenous retroviruses (HERVs) reverse transcribe the mutated transcripts into DNA species that are then inserted back into the genomes of the host neurons. The resulting transcripts, translated into mutant proteins, are prone to aggregation, as occurs in familial or sporadic forms of Alzheimer’s disease (AD). Theoretically, HERV RTs could be blocked by RT inhibitors (RTIs) that are currently in widespread use for the treatment of HIV infection. The hypothesized clinical significance of the purported pathways is that: (1) since almost all people with HIV (PWH) are taking RTIs, the mechanism could explain the paucity of reported cases of AD in PWH; and (2) these already-approved RTIs could be offered more broadly for AD prevention in people who do not have HIV infection. Our objective was to test this hypothesis in the clinical setting by studying cases of PWH treated for many years with RTIs who nevertheless demonstrate substantial cerebral Alzheimer-type amyloidosis. We also report the prevalence of amyloid plaque pathology among an autopsy cohort of PWH who were previously characterized for amyloid pathology for a prior study (Soontornniyomkij et al. 2012; Umlauf et al. 2019). Cases were chosen to highlight in a case series because they met the following criteria: (1) showed substantial amyloid pathology either at autopsy or via cerebrospinal fluid biomarker levels and (2) their clinical data suggest clinical significance of this pathology either through Alzheimer’s-like cognitive/functional decline or meeting clinical criteria for an AD diagnosis (McKhann et al. 2011). To reinforce our findings in this case series, we examined the prevalence and correlates of amyloidosis in a larger autopsy cohort. This larger cohort study represents the 250 cases at UCSD with pathological and clinical information available, including APOE status, *and* who died after 2006, allowing for at least several years of virally suppressive NRTI-containing ART was 250. This should not be considered a representative sample of PWH. The sample was one of convenience and is subject to numerous biases, including selection and referral biases.

## Materials and Methods

### Clinical Evaluations

Patients were seen in the context of a prospective study of the neurological consequences of HIV infection at the HIV Neurobehavioral Research Program (HNRP) at the University of California, San Diego (UCSD) and at the UCSD outpatient Neurology Clinic between 2009 and 2019. Written informed consent was obtained from all participants in the study to participate in research, including autopsy. All underwent serial neuropsychological evaluations that applied demographic corrections to raw test data (Heaton and Iudicello 2018) and neurological examinations according to previously published methods. Two had neuropathological evaluations and one was assessed clinically for AD by cerebrospinal fluid (CSF) analysis. HIV RNA levels were measured using standard PCR-based clinical assays, and blood CD4 + T lymphocyte counts by flow cytometry. Levels of phosphorylated-Tau, Total-Tau and Aβ42, were assayed by ELISA using the ADmark Alzheimer’s Evaluation and the Aβ42/total tau index (ATI) was calculated. Results were reported as associated with AD using ATI < 1.0 and pTau > 61 pg/ml as thresholds.

#### Autopsy Brain Specimens

Formalin-fixed, paraffinembedded (FFPE) autopsy brain specimens were available from two cases for the analysis of presence of markers of AD-like neuropathology: β-amyloid (Aβ) deposition and phosphorylated Tau (p-Tau) positive neurofibrillary tangles. The available paraffin blocks included the following regions: frontal cortex (Brodmann area 8), superior temporal cortex, hippocampus, anterior striatum, and posterior striatum. For the larger autopsy cohort, we constructed a semiquantitative rating reflecting the distribution of amyloid staining where focal/occasional = 1; widespread/abundant = 2. Ratings were independent of whether staining was diffuse versus consolidated plaques.

### Immunohistochemistry

The primary antibodies were mouse monoclonals against beta amyloid (4G8, Biolegend, San Diego, CA, USA, #800,708), and phospho-tau (AT8, Pierce Biotechnology, Rockford, IL, USA, #MN1020). Five-µm thick formalin-fixed, paraffin-embedded tissue sections were deparaffinized with histoclear and rehydrated through a graded ethanol series and water. Antigen retrieval was carried out in an autoclave at 121 °C for 10 min with 88% formic acid for beta amyloid and 0.01 M sodium citrate/ 0.05% Tween 20 buffer (pH 6) for phospho-tau. The tissue sections were treated for 20 min with 0.3% hydrogen peroxide/PBS to quench endogenous peroxide activity. Following 1-h incubation at room temperature with the primary antibody diluted in Dako antibody diluent (DakoCytomation, Carpinteria, CA, USA). The immunoreactive signals were detected with the peroxidase-labeled polymer-conjugated horse anti-mouse IgG secondary antibody (ImmPRESS -VR, Vector Laboratories, 40 min at room temperature) and NovaRED substrate (ImmPACT, Vector Laboratories, 3 min at room temperature). All the sections were counterstained with hematoxylin and mounted with Cytoseal 60 (Richard-Allan Scientific, Waltham, MA, USA). For the negative control, the primary antibody was omitted.

## Discussion and Evaluation

### Case #1

This was a white participant in their 80s with HIV with a high school diploma. At the time of clinical evaluation, estimated duration of HIV disease was 18 years, nadir CD4 was 37, current CD4 653, and plasma HIV RNA was undetectable on dolutegravir, the nucleoside reverse transcriptase inhibitor (NRTI) abacavir and lamivudine. The participant took ARVs that included at least one NRTI for 16 years. With the exception of hyperlipidemia, osteopenia and a malignancy (not specified) in remission, they reported no major vascular, pulmonary, hepatic or other comorbidities. Concomitant medications were alendronate, aspirin and atorvastatin. They had no history of substance use disorders and reported no family history of AD.

The participant underwent three neuropsychological evaluations within a two-month period before their death eight months later. Across all evaluations, they reported dependence on others to accomplish important activities of daily living. They denied significant depression but reported fatigue and generalized weakness (Profile of Mood States vigor/activation subscale). Consistently across all evaluations, their performance indicated mild global neurocognitive impairment. Domain-specific neurocognitive performance demonstrated mild-to-moderate verbal impairment, mild executive function impairment and normal motor function. Speed of information processing performance vacillated between borderline impaired (1st evaluation), normal/average (2nd evaluation), and below average (3rd evaluation). Working memory performance was in the borderline impaired range in the first two evaluations, but normal/average at the third evaluation. Even within this short time span, deterioration was observed in memory function. Their first and second evaluations indicated below average learning and recall performance, whereas their third and final evaluations indicated borderline impaired learning performance and mildly impaired recall performance. In parallel, using an amnestic mild cognitive impairment (aMCI) diagnostic method that emphasizes impairments in recognition, the participant’s memory performance was classified as low aMCI risk at their first and second evaluations, but high aMCI risk at the third evaluation (Sundermann et al. 2020). Small declines (~ 2 T-score units) in both learning and recall performance from the first two evaluations to the third contributed to the overall deterioration in memory performance. In contrast, recognition performance was consistent across evaluations with verbal recognition in the impaired (~ 2 SD below the normative mean) range and visual recognition in the normal/below average range. The Global Deficit Score was 0.76, indicating mild-moderate overall impairment, with domain deficit scores indicating moderate impairment in learning, recall and verbal fluency (Table 1).

**Fig. 1:**
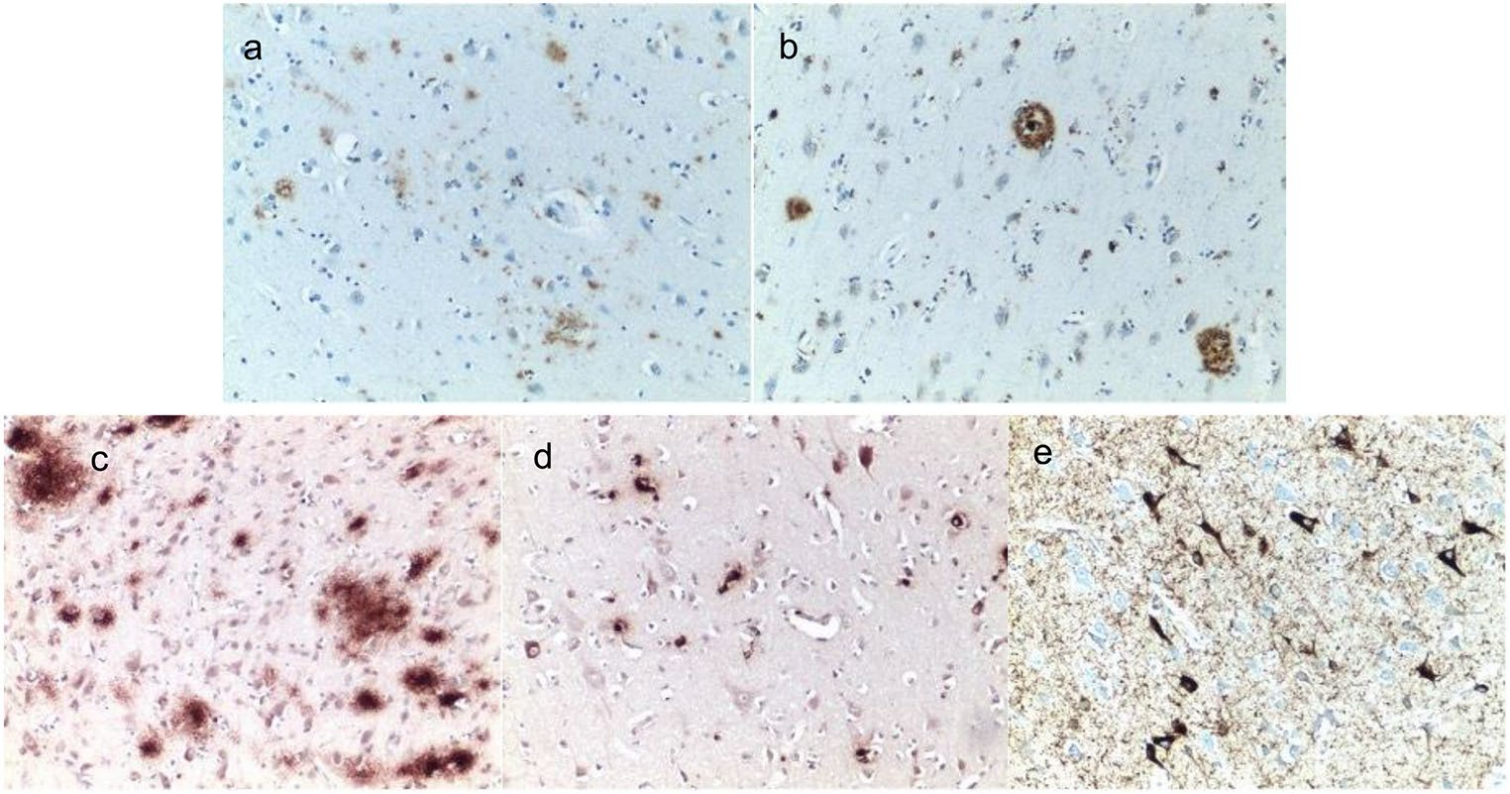
Immunostaining for amyloid and pTau: Frequent diffuse (**a**) and occasional senile plaques (**b**) were found in the frontal cortex and amygdala for Case #1. Diffuse amyloid plaques were found in in the frontal cortex (**c**), intraneuronal amyloid was found in the superior temporal cortex (**d**), and pTau neurofibrillary tangles and threads were found in the hippocampus for Case #2. Original magnification 10X; counterstaining with hematoxylin

*Neuropathology for Case #1*. Gross examination of the brain at autopsy showed atherosclerosis of the right middle cerebral artery with 80% stenosis. Microscopic examination of H&E slides showed neocortical neuritic senile plaques, amyloid angiopathy, arteriosclerosis and arteriolosclerosis in subcortical white matter, and vascular mineralization in left globus pallidus. Only a few CA1 neurons displayed granulovacuolar degeneration. Immunohistochemistry showed amyloid deposition predominantly as diffuse plaques with occasional consolidated plaques, and in a few instances intraneuronal staining (Fig. 1a, b).

### Case #2

This was a white participant in their 60s with HIV who had obtained a bachelor’s degree and worked in a high skill level position according to the International Standard Classification of Occupations (ISCO). The estimated duration of HIV disease was 20 years. The nadir CD4 was 143, current CD4 411, plasma viral load 981 copies/mL on atazanavir, tenofovir and emtricitabine. They had taken NRTIs for 17 years. Comorbidities were osteoarthritis, osteoporosis, hypothyroidism and hyperlipidemia. The participant had received appropriate treatment for primary syphilis in 2008 and reported a history of optic neuritis of undetermined etiology for many years. Concomitant medications were atorvastatin, escitalopram, prednisone, gabapentin, tamsulosin and levothyroxine. They had required use of a walker and had clumsy hands for many years. They endorsed mild-to-moderate depressed mood, severe fatigue and general confusion. They reported that their mother and maternal grandmother had AD.

The participant completed four neurocognitive evaluations in a two-year period (2003–2005) before death in 6/2006. Across all evaluations, they reported dependence on others to accomplish important daily activities. Global neurocognitive performance was deemed moderately impaired in the first three evaluations and deteriorated to severe impairment in the final evaluation. Verbal and working memory performance also showed decline over time from average or below average at the first and second evaluations to mild impairment at the 3rd and moderate impairment at the final evaluation. Speed of information processing was borderline impaired at the initial evaluation and then also showed deterioration to mild-to-moderate or moderate impairment at the 2nd and 3rd evaluations, culminating in severe impairment at the final evaluation. Executive function was the most consistently impaired domain throughout follow-up with moderate or moderate-to-severe impairment at the 1st through 3rd evaluations and deteriorating even further to severe impairment at the final evaluation. Motor performance vacillated within the range of mild-to-moderate to moderate-to-severe in the first three evaluations and deteriorated to severe impairment in the final evaluation. Again, using a method emphasizing recognition memory impairment, the participant was classified as high aMCI risk at all evaluations. Whereas learning performance showed a gradual decline from mild impairment (1st evaluation) to mild/moderate (2nd evaluation) to moderate impairment (3rd and 4th evaluations), recall performance showed a steeper decline from borderline impaired at the 1st evaluation to moderate-to-severe impairment in the 2nd evaluation and 4th evaluations with a brief improvement to mild impairment at the 3rd evaluation. In contrast, recognition performance showed stable impaired performance (~ 2 to 3 SD below the normative mean) across all evaluations (Table 1). They died in June 2006.

*Neuropathology for Case #2*. Microscopic examination of the H&E slides showed scattered diffuse plaques. Neurofibrillary degeneration of mild degree was noted within the hippocampus and amygdala. Immunohistochemistry examination showed abundant amyloid deposition as diffuse plaques in the frontal cortex (Fig. 1c), with occasional intraneuronal localization in the superior temporal cortex (Fig. 1d), and prominent neurofibrillary tangles in the hippocampus (Fig. 1e), immunohistochemistry for HIV gp41 envelope protein was negative.

### Case #3

This was a non-white participant in their 70s with HIV who had completed 18 years of formal education. At the time of clinical presentation, they had lived with HIV for 34 years. Their nadir CD4 + lymphocyte count was 259 cells/uL, and the current CD4 was 409 with an undetectable plasma viral load on dolutegravir, emtricitabine and tenofovir alafenamide. They had taken nucleoside NRTIs continuously for 12 years. The participant had a history of hypertension, hyperlipidemia and Type II diabetes mellitus, did not smoke tobacco and denied use of alcohol or illicit drugs with no history of substance use disorders. Concomitant medications included amlodipine, aspirin, benazepril, ezetimibe, metformin, and sitagliptin. They denied significant mood symptoms and reported no family history of AD.

In May 2016, the participant presented to the Neurology clinic complaining of transient lapses of attention without functional impairment. Mini-Mental State Examination (MMSE) score was 29/30. A brain MRI showed extensive periventricular T2 white matter hyperintensities and small bilateral basal ganglia lacunes. A carotid ultrasound demonstrated atherosclerosis, but no hemodynamically significant stenosis. A working diagnosis of mild cognitive impairment due to cerebral small vessel disease was made. In September 2016, they reported worsening memory difficulties without functional impairment, and was started on rivastigmine. By October 2016, the MMSE had dropped to 27. They were then referred to a research study for neurocognitive testing which showed high premorbid intellectual ability. The participant was classified as high aMCI risk based on impaired memory recall and recognition performance and reported dependence on others to accomplish important daily activities. At subsequent evaluations they denied dependence in activities of daily living, but we believe that this is likely due to lack of insight into their own neurocognitive deficits, since informant history indicated increasing dependence in all instrumental activities of daily living. By July 2017, the score on the Montreal Cognitive Assessment (MOCA) was 24/30. In May 2018 the patient’s family members expressed serious concerns about the participant’s memory problems and safety issues.

**Fig. 2:**
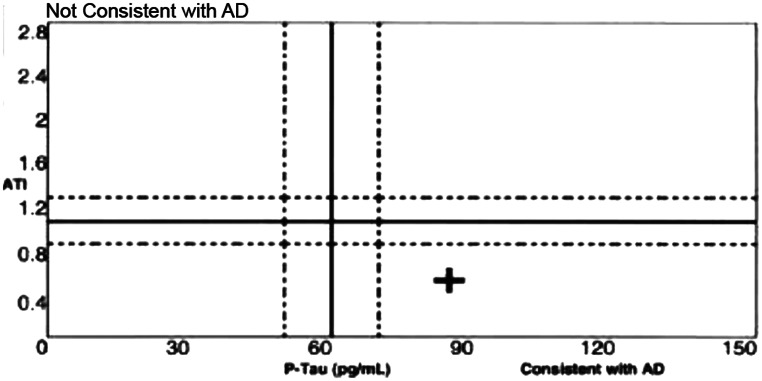
Plot of phospho-Tau versus Aβ42/total tau index for Case #3 showing values falling within the expected quadrant for Alzheimer’s disease (AD)

The MOCA score had dropped to 17, and a diagnosis of probable Alzheimer’s Disease with comorbid cerebral small vessel disease was made. Repeat comprehensive neuropsychological testing showed overall moderate global impairment; the greatest deficits were in speed of information processing, verbal fluency, verbal learning and delayed recall, and executive functioning. Despite the concerns of the participant’s family members, they were still driving and handling finances. Repeat neurocognitive testing showed global moderate-to-severe impairment. CSF showed Aβ42 406.65 pg/ml (reference range > 500 ng/L), T-Tau 531.1 pg/mL (reference range < 350 pg/ml) and phosphorylated Tau181 84.2pg/mL (reference range < 61) with an ATI > 0.8, consistent with AD (Fig. 2).

The patient completed four annual neurocognitive evaluations in the early 1990s when they were in their late forties, and then again between 2016 and 2019 when they were in their 70s. Their global neurocognitive performance during the four evaluations in the early 1990s fluctuated between average (1991), borderline impaired (1990 and 1996) and mild-to-moderate impairment (1995). When some degree of global impairment was evident, it was driven mostly by mild-to-moderate impairment in working memory, speed of information processing and executive function. In contrast, verbal skills, learning, recall and motor performance were classified as normal throughout the early evaluations except for borderline impaired performance at their last evaluation (Table 1).

**Fig. 3:**
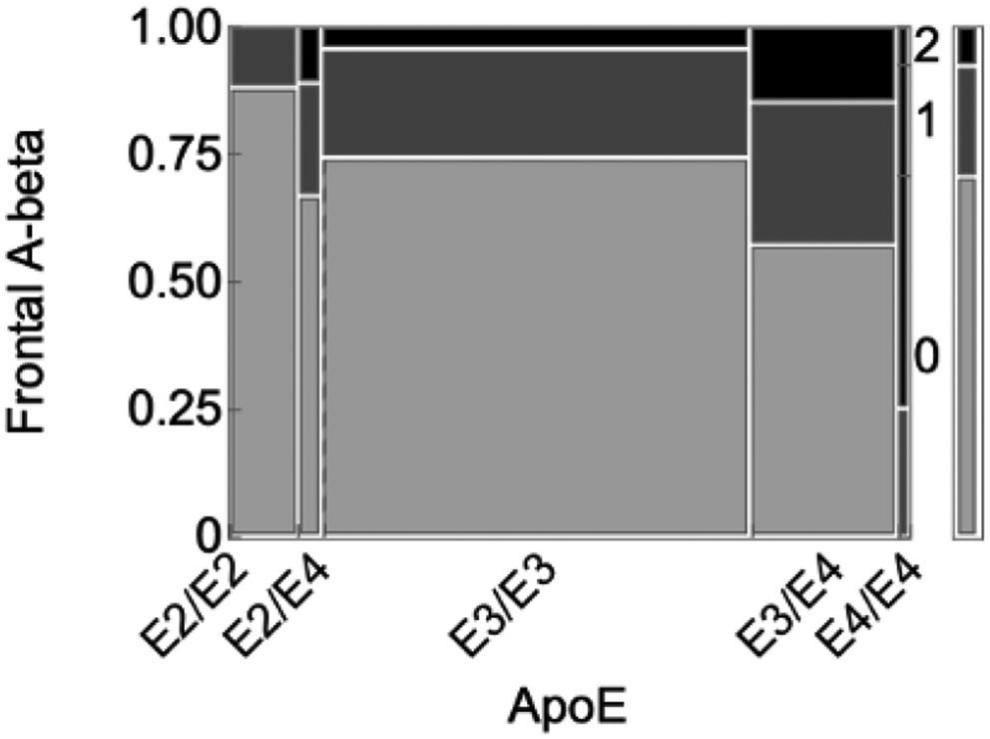
Semiquantitative amyloid stain rating according to ApoE genotype. Increasing E4 alleles were associated with higher amyloid ratings

**Table 1:** Case summary of neurocognitive (at last neurocognitive evaluation) and neuropathology characteristics

**Neurocognitive Characteristics**											
	ADL dependent	Global cognitive impairment	Verbal impairment	Executive function impairment	Learning impairment	Recall impairment	Information processing speed impairment	Working memory impairment	Motor impairment	aMCI risk diagnosis	Memory deterioration
Case #1	Yes	Mild/ moderate	Mild/ moderate	Mild	Borderline	Mild	Yes	No	No	High	Yes
Case #2	Yes	Moderate/ severe	Mild/ moderate	Moderate/ severe	Mild/ moderate	Moderate/ severe	Yes	Yes	Yes	High	Yes
Case #3	Yes	Moderate/ severe	Mild	Mild/ moderate	Borderline	Borderline	Yes	Yes	No	High	Yes
**Neuropathology Characteristics**											
	Amyloid deposition	Neuro-fibrillary tangles	atherosclerosis	arteriosclerosis							
Case #1	Yes	Yes	Yes	Yes							
Case #2	Yes	Yes	Yes	Yes							

The larger autopsy cohort comprised 250 PWH brains from PWH that underwent neuropathological characterization of frontal lobe Aβ and ApoE genotyping as part of a prior study (Umlauf et al. 2019). The mean (SD) age at death was 47.4 (9.22) years; 17.6% were female; 21.6% were Black, 22.8% Hispanic, 52.0% Non-Hispanic White, and 3.60% other race/ethnicities; mean (SD) education 12.5 (2.87) years. 169 (67.6%) had semiquantitative amyloid ratings of 0, sixty (24%) of 1 (focal/occasional), and 21 (8.4%) of 2 (widespread/abundant). The distribution of ApoE genotypes was as follows: E2/E2 1 (0.40%), E2/E3 25 (10.0%), E2/E4 9 (3.60%), E3/E3 157 (62.8%), E3/E4 54 (21.6%), E4/E4 4 (1.60%). As seen in Fig. 3, higher amyloid ratings were associated with the presence of the ApoE E4 allele, the strongest genetic risk factor for sporadic AD (Pearson χ^2^ = 39.7, *p* = 1.88e-5). Poorer ante-mortem neurocognitive performance was not associated with higher amyloid burden (*p* = 0.580); however, the probability of HAND was increased in the presence of Aβ plaques when limiting to APOE E4 carriers (OR = 30.00, 95% CI = 1.41-638.63, *p* = 0.03) (Umlauf et al. 2019). This suggests that, in the context of higher AD genetic risk, amyloid burden has greater clinical significance among PWH. The amyloid ratings in those who took NRTIs as part of their antiretroviral regimens (*n* = 160, 58.6%; amyloid rating 0, 69.4%; amyloid rating 1, 20%; amyloid rating 2, 10.6%) did not differ from those who did not take NRTIs (amyloid rating 0, 72.6%; amyloid rating 1, 23.9%; amyloid rating 2, 3.54%; *p* = 0.178).

## Conclusions

Here we report three virally suppressed PWH who had clinical evaluations in the 7th, 8th and 9th decades of life and who had been treated for many years with RTIs. Two patients showed substantial cerebral amyloid deposition at autopsy with evidence of worsening neuropsychological impairment over time, and a third met clinical criteria for AD based on a typical clinical course and CSF biomarker profile with reduced Aβ42 concentrations.

The neuropsychological tests described here were conducted as part of a standard research protocol and were not a formal clinical evaluation, as no formal clinical interview was conducted. With that said, the neuropsychological tests and self-report instruments (e.g., Activities of Living Questionnaire, Beck Depression Inventory-II) used in the case studies above are consistent with the methods that are typically used in the clinical assignment of diagnoses such as Alzheimer’s Disease.

When interpreting results, it is important to consider that our sample is not representative of the general population of PWH. The sample was one of convenience and is subject to numerous biases, including selection and referral biases. Generalizability of our findings is also limited due to the fact that the sample was mostly male and an autopsy cohort characterized by advanced medical morbidity. However, with the larger cohort we hope to provide context for comparison (Table 2). Although a prospective study would be ideal, a randomized clinical trial in offering RTIs to some patients and not others is not feasible. Moreover, long-term study prospective studies are difficult because they require early identification of individuals at risk of Alzheimer’s Disease who would then be followed for many years to assess for conversion to AD.

**Table 2:** Characteristics of the larger cohort study of PWH

**Demographics**	
Education in years, *M* (SD)	12.5 (2.9)
Male, n (%)	70.7%
Race/Ethnicity, n (%)	
Black/African-American	54 (21.6%)
Hispanic	57 (22.8%)
White	130 (52.0%)
Other	9 (3.6%)
ApoE-ε4 allele carrier, n (%)	67 (26.8)
**HIV Disease Characteristics**	
Antemortem HAND dx, n (%)	
Asymptomatic Neurocognitive Impairment	33 (13.2%)
Mild Neurocognitive Disorder	58 (23.2%)
HIV-associated Dementia	44 (17.6%)
**Amyloid Plaque Ratings**	
0 (not present), n (%)	177 (70.8%)
1 (focal/occasional), n (%)	54 (21.6)
2 (widespread/abundant), n (%)	19 (7.6)

*Notes*. PWH = people with HIV, ART = antiretroviral therapy, HAND = HIV-associated neurocognitive disorders

Despite their relatively young age, in the larger cohort of autopsied PWH, cerebral amyloidosis was common. Among PWH on RTIs, the prevalence of cerebral amyloidosis was statistically not different from those not taking RTIs, arguing against a protective role of RTIs in amyloid deposition. Umlauf and colleagues found that amyloid burden was related to poorer antemortem neurocognitive performance, only among ApoE E4 carriers (Umlauf et al. 2019).

Beyond the questionable role of RTIs in mitigating cerebral amyloidogenesis, these drugs are not without toxicities. The primary mechanism of NRTI toxicity is mitochondrial toxicity, energy depletion, and oxidative stress, which have been demonstrated both in vitro and in vivo (Lewis et al. 2003; Kohler and Lewis 2007; Nooka and Ghorpade 2018). The major clinical toxicities are renal insufficiency (Rodriguez-Nóvoa et al. 2010; Scherzer and Shlipak 2015) and bone loss (Barber et al. 2016). Moreover, the absence of a large bolus of AD among PWH is likely the result of the relatively small number of PWH who are in their 70s and beyond when sporadic AD typically manifests. Based on data showing that the lifespan of PWH is approaching that of the general population, we expect to see large increased in the number of PWH in their seventies and beyond. Unfortunately, this will likely mean more PWH evidencing AD and other related dementias. Research to identify these cases and understand mechanisms of, and treatment for, AD, both in and out of the context of HIV, remain sorely needed.

The vast majority of current regimens contain NRTIs, the most common of which are tenofovir, emtricitabine, abacavir and lamivudine. Tenofovir causes renal and bone toxicity (Woodward et al. 2009). Of particular interest in the context of brain disease, numerous studies show neurotoxicities of NRTIs. Thus, in vitro studies showed loss of microtubules-associated protein type 2 (MAP-2) immunostaining, reflecting dendritic simplification, impaired acute and delayed intra-cellular calcium responses to glutamate and depletion of neuronal mitochondrial membrane potentials (Robertson et al. 2012). Tenofovir reduced expression of mitochondrial transcription factor A (TFAM), essential for the maintenance of mitochondrial DNA, in mouse hippocampus (Fields et al. 2019). A regimen containing tenofovir and emtricitabine increased the generation of toxic reactive oxygen species (ROS) in Eco-HIV-infected mouse neural progenitor cells (NPCs) (Velichkovska et al. 2019). In vitro, NRTIs cause endothelial cell dysfunction and impaired blood-brain barrier permeability (Faltz et al. 2017; Bertrand et al. 2021).

Others have also reported presence of amyloid in the brain of PWH (Esiri et al. 1998; Izycka-Swieszewska et al. 2000; Nebuloni et al. 2001; Gelman and Schuenke 2004; Green et al. 2005; Achim et al. 2009; Soontornniyomkij et al. 2012; Turner et al. 2016) including newer PET studies using [18 F]Florbetaben. Moreover, numerous autopsy studies showed cerebral Aβ deposits and amyloid angiopathy in the brains of individuals dying with HIV (Esiri et al. 1998; Izycka-Swieszewska et al. 2000; Nebuloni et al. 2001; Gelman and Schuenke 2004; Green et al. 2005; Achim et al. 2009; Soontornniyomkij et al. 2012). However, we are the first to carefully look at the relationship of cerebral amyloidosis to long-term NRTI exposure.

In sum, it is premature to propose to treat or prevent Alzheimer’s disease using NRTIs. Although FDA approved, their toxicities are substantial. Evidence that they protect from cerebral amyloidogenesis is unconvincing.

## Data Availability

Anonymized data not published within this article will be made available by request from any qualified investigator.
